# Late Gastrointestinal Toxicity After Dose-Escalated Conformal Radiotherapy for Early Prostate Cancer: Results From the UK Medical Research Council RT01 Trial (ISRCTN47772397)

**DOI:** 10.1016/j.ijrobp.2009.05.052

**Published:** 2010-07-01

**Authors:** Isabel Syndikus, Rachel C. Morgan, Matthew R. Sydes, John D. Graham, David P. Dearnaley, MRC RT01 collaborators

**Affiliations:** ∗ Clatterbridge Centre for Oncology, Bebington, United Kingdom; † Cancer Group, MRC Clinical Trials Unit, London, United Kingdom; ‡ Taunton and Somerset NHS Foundation Trust, Musgrove Park Hospital, Taunton, United Kingdom; § Institute of Cancer Research and Royal Marsden Hospital, Sutton, United Kingdom

**Keywords:** Prostate cancer, Conformal radiotherapy, Dose escalation, Late gastrointestinal toxicity, Phase III trial

## Abstract

**Purpose:**

In men with localized prostate cancer, dose-escalated conformal radiotherapy (CFRT) improves efficacy outcomes at the cost of increased toxicity. We present a detailed analysis to provide further information about the incidence and prevalence of late gastrointestinal side effects.

**Methods and Materials:**

The UK Medical Research Council RT01 trial included 843 men with localized prostate cancer, who were treated for 6 months with neoadjuvant radiotherapy and were randomly assigned to either 64-Gy or 74-Gy CFRT. Toxicity was evaluated before CFRT and during long-term follow-up using Radiation Therapy Oncology Group (RTOG) grading, the Late Effects on Normal Tissue: Subjective, Objective, Management (LENT/SOM) scale, and Royal Marsden Hospital assessment scores. Patients regularly completed Functional Assessment of Cancer Therapy--Prostate (FACT-P) and University of California, Los Angeles, Prostate Cancer Index (UCLA-PCI) questionnaires.

**Results:**

In the dose-escalated group, the hazard ratio (HR) for rectal bleeding (LENT/SOM grade ≥2) was 1.55 (95% CI, 1.17–2.04); for diarrhea (LENT/SOM grade ≥2), the HR was 1.79 (95% CI, 1.10–2.94); and for proctitis (RTOG grade ≥2), the HR was 1.64 (95% CI, 1.20–2.25). Compared to baseline scores, the prevalence of moderate and severe toxicities generally increased up to 3 years and than lessened. At 5 years, the cumulative incidence of patient-reported severe bowel problems was 6% vs. 8% (standard vs. escalated, respectively) and severe distress was 4% vs. 5%, respectively.

**Conclusions:**

There is a statistically significant increased risk of various adverse gastrointestinal events with dose-escalated CFRT. This remains at clinically acceptable levels, and overall prevalence ultimately decreases with duration of follow-up.

## Introduction

Patients with low or intermediate risk prostate cancer have improved disease outcome in terms of local control, biochemical control, and disease-free survival if they are treated with dose-escalated rather than “standard-dose” conformal radiotherapy (CFRT) according to five randomized controlled trials from North America, The Netherlands, and the UK. However, dose-escalated radiotherapy has more late gastrointestinal effects and not bladder toxicity (1–7). Long-term survival after treatment is the rule (9); thus, patients who develop significant side effects from radical radiotherapy may have to live with them for a prolonged period of time.

The Medical Research Council (MRC) RT01 trial was a large, multicenter, randomized controlled trial in men with localized prostate cancer, who were randomized to receive either standard (64-Gy) or dose-escalated (74-Gy) CFRT. All patients received neoadjuvant hormone therapy. The design, patient, and treatment data and toxicity and early outcome results (6–8) have been presented in detail: at 5 years, the cumulative incidence for RTOG grade ≥2 bowel toxicity was 24% for the standard-dose group and 33% for the escalated-dose group (6–8). We report here the detailed analysis of late bowel toxicity.

## Methods and Materials

The MRC RT01 trial recruited men with T1b to T3a, N0, M0 prostate cancer, with a prostate-specific antigen concentration of ≤50 ng/ml. Patients received androgen suppression for 3 to 6 months before radiotherapy started. Patients were randomized to receive either 64 Gy in 32 fractions (standard group) or 74 Gy in 37 fractions (escalated group) (6, 7). The dose to rectum, small bowel, and anal canal was not routinely calculated during the planning process, and no attempt was made to reduce normal tissue doses. Patients were assessed for toxicities before starting androgen suppression, before starting radiotherapy, and at 6, 12, 18, and 24 months and annually thereafter. Only events reported at least 6 months after starting radiotherapy were deemed late events. Events occurring earlier were considered early toxicities, and descriptions have been published previously (6).

### Toxicity assessment

Three physician-completed toxicity assessments were used: the Radiation Therapy Oncology Group (RTOG) (10) scale, the Royal Marsden Hospital (RMH) scale (5), and the Late Effects on Normal Tissue, Subjective, Objective, Management (LENT/SOM) questionnaire (11). The RTOG scale used matched that from the pilot study, measuring five bowel toxicities separately (10). It was not completed at preradiotherapy assessments. The RMH scale consists of five questions assessing the commonest bowel side effects after pelvic radiotherapy. The LENT/SOM instrument divides toxicity into subjective symptoms reported by patients (*e.g.,* pain), objective symptoms (*e.g.*, diarrhea), and medical intervention required to control toxicity (*e.g.,* medications or transfusions). Additionally, patients completed UCLA Prostate Cancer Index (UCLA-PCI) (12) and Functional Assessment of Cancer Therapy-Prostate (FACT-P) (13) scales to assess quality of life. These questionnaires asked 27 questions about bowel symptoms.

Responses to RTOG questions were categorized as grades 0, 1, 2, and ≥3 for this analysis. LENT/SOM grades 0 to 1 were broadly equivalent to RTOG grade 0; therefore, LENT/SOM grades 2, 3, and 4 translated to RTOG grades 1, 2, and ≥3. However, LENT/SOM diarrhea grade 1 was similar to RTOG grade 1 severity, and so it was categorized as 0, 1, 2, and ≥3 (see Table E1 in the supplementary material). The RMH, UCLA-PCI, and FACT-P questions did not have standard combined scales, so each variable from these questionnaires was considered individually, with response categories designated mild, moderate, or severe (see Table E1 in the supplementary material). The composite RTOG score has previously been reported (7) and is not detailed additionally here.

### Statistical analyses

Analyses used a two-sided, 5% significance level on an intention-to-treat basis, with randomized patients analyzed according to their allocated treatment group. All analyses were performed using Stata (version 9) software.

Standard time-to-event (survival analysis) methodology was used to assess the first reported incidence of each severity level of each toxicity endpoint. Events were timed from the start of radiotherapy, and the differences between the treatment groups were tested using the log-rank test. Relative risks of these unwanted effects according to treatment are summarized using hazard ratios (HR) with 95% confidence intervals (CI) from Cox regression models. All comparisons are expressed relative to the standard group; so an HR value of <1.00 indicates lower risk of toxicity in the escalated-dose group.

The prevalence of each grade of toxicity at each time point (including pretreatment) is presented in tables and as stacked bar graphs; no formal statistical tests have been performed with these data. These graphs present all data collected between 10% below and 30% above a certain time point in days, to a maximum of 6 months. Only patients for whom all five assessments were available for a certain time point were included in these analyses. Data for these analyses were frozen in March 2007; thus, this work is based on the same dataset as the efficacy results paper, which contained an overview of toxicity results (7).

## Results

Between January 1998 and December 2001, 843 men were randomized to MRC RT01: 422 men were allocated to the escalated-dose arm, and 421 men were allocated to the standard arm. Compliance with the allocated treatment was excellent, with 95% of the standard and 97% of the escalated patients receiving the correct radiotherapy dose. At the preradiotherapy time point, all five forms were returned for 702/843 (83%) patients. Compliance with all scales at 6, 12, and 24 months was 605/764 (79%) patients, 645/790 (82%) patients, and 592/762 (78%) patients, respectively, and was 306/592 (52%) patients at 5 years.

### Rectal bleeding

**Fig. 1:**
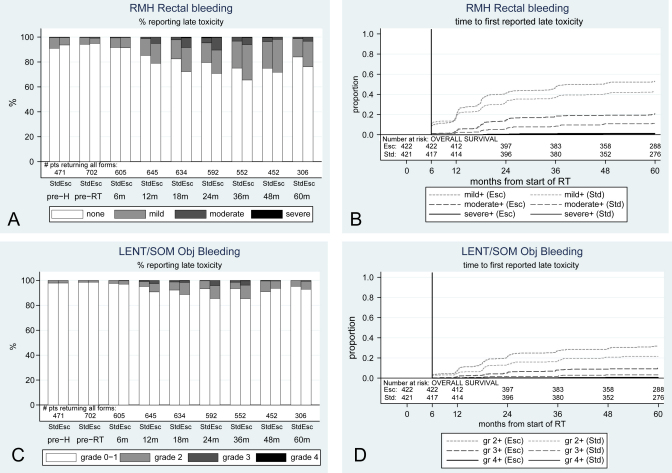
Prevalence and cumulative incidence of rectal bleeding. (A, B) RMH; (C, D) LENT/SOM. Obj = objective; Std = standard arm, 64 Gy; Esc = escalated arm, 74 Gy; m = month; gr = grade; pre-H = prehormonal therapy; pre-RT = preradiation therapy.

Clinicians recorded low baseline levels of rectal bleeding for 34/702 (5%) patients having mild and 4/702 (1%) patients having moderate bleeding on the RMH scale, and 10/702 (1%) patients having grade 2 on the LENT/SOM scale. Rectal bleeding was the most commonly reported bowel toxicity, with a cumulative incidence by 5 years for mild-or-worse bleeding on the RMH scale at 43% (170 patients) for the standard group and 53% (211) for the escalated group (Table 1). Escalated patients were significantly more likely to report mild-or-worse bleeding and moderate-or-worse bleeding, according to the RMH scale (mild-or-worse HR = 1.32; 95% CI, 1.08-1.61; moderate-or-worse HR = 1.97; 95% CI, 1.37-2.84). They were also significantly more likely to report grade ≥2 and grade ≥3 bleeding on the LENT/SOM objective scale (grade ≥2 HR = 1.55; 95% CI, 1.17-2.04; grade ≥3 HR = 3.12; 95% CI, 1.63-6.01). Severe bleeding was rare; by 5 years from starting radiotherapy, just 3 standard and 5 escalated patients reported the most severe RMH toxicity; 3 and 1 patients reported the most severe LENT/SOM toxicity. Escalated patients required more medical interventions for bleeding (LENT/SOM bleeding management), but the difference was not statistically significant: by 5 years, 17 escalated patients had required ≥1 transfusion or laser treatment, 8 escalated patients had required more regular transfusions, and 4 escalated patients required surgery compared with 9, 3, and 1 patients in the standard arm, respectively. After 6 months, the escalated arm patients had a higher prevalence of any bleeding toxicity at almost every time point (Fig. 1). Incidence peaked around 3 years after radiotherapy, and at 5 years, levels remained higher than those at baseline in both arms. The slowly rising overall incidence was due to new reports of bleeding up to 5 years postradiotherapy. For example, between 2 and 5 years after radiotherapy, 28 occurrences of LENT/SOM grade ≥2 objective bleeding were reported by patients in the standard arm who had not previously reported this symptom (compared to 83 reports in total over 5 years) and 35 occurrences were reported in the escalated arm (compared to 121 over 5 years). The prevalence of bleeding decreased from year 3 to year 5.

### Diarrhea and frequency

Incidence of diarrhea or loose stools was low preradiotherapy according to physician-reported scores, with 38/702 (5%) patients reporting mild symptoms and 4/702 (1%) patients reporting moderate symptoms on the RMH questionnaire. Patients reported higher levels of problems. Mild diarrhea was frequently reported in both treatment arms throughout the observation period, both before and after radiotherapy, with 341, 16, and 6 of 702 (49%, 2%, 1%, respectively) patients reporting mild, moderate, and severe loose stools, respectively on the UCLA-PCI preradiotherapy questionnaire; the corresponding numbers for abdominal cramps on the same questionnaire were 122 (18%), 38 (5%), and 17 (2%) of 702 patients, respectively.

**Fig. 2:**
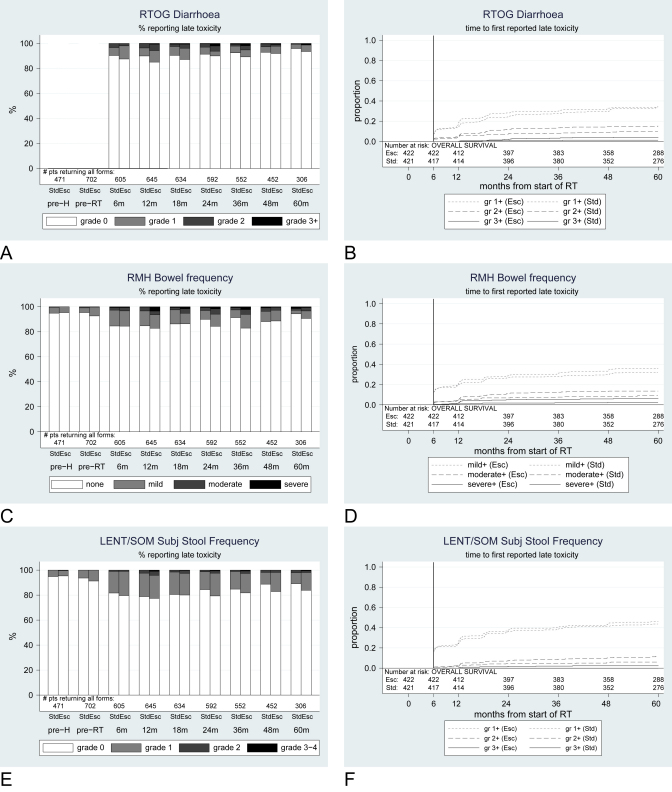

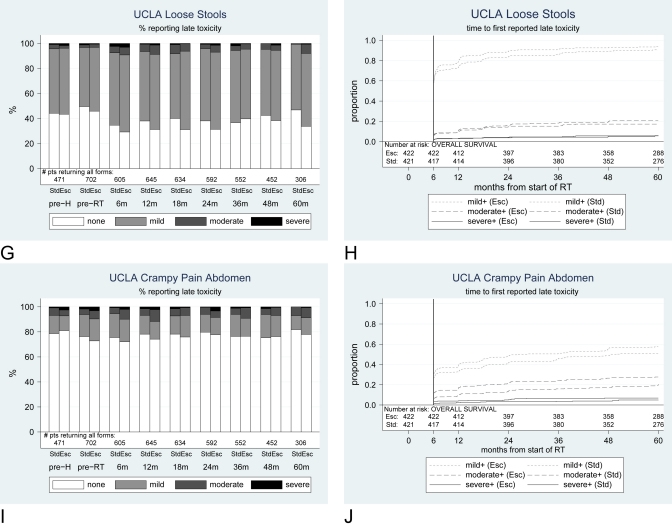
Prevalence and cumulative incidence of diarrhea. (A, B) RTOG; (C, D) RMH; (E, F) LENT/SOM; (G, H) UCLA-PCI; (I, J) abdominal pain, UCLA-PCI. Subj = subjective; Std = standard arm; Esc = escalated arm; m = month; gr = grade; pre-H = prehormonal therapy; pre-RT = preradiation therapy.

Moderate-to-severe diarrhea was more commonly reported by physicians in the escalated arm, and this difference was statistically significant for several items in this category (Table 2), in particular, RTOG grade ≥2 (HR = 1.59; 95% CI, 1.07-2.35) and grade ≥3 (HR = 3.04; 95% CI, 1.21-7.67). However, grade ≥3 diarrhea was not common, with only 5 standard and 16 escalated arm patients reporting such severe symptoms by 5 years. Once more, escalated patients required more medical interventions according to LENT/SOM management of tenesmus/stool frequency, but the difference was not significant: the cumulative incidence by 5 years of receiving medication more than twice-weekly to control diarrhea was 6% (*n* = 23 patients) for standard and 8% (*n* = 32 patients) for escalated patients, while 4 and 9 patients, respectively, had needed multiple drugs daily. No patients reported surgical procedures for diarrhea. Physician-reported prevalence of diarrheal symptoms lessened once patients reached about 3 years from radiotherapy, although this was less clear for patient-reported outcomes. At most time points, prevalence was higher in the escalated arm than in the standard arm (Fig. 2).

### Proctitis

**Fig. 3:**
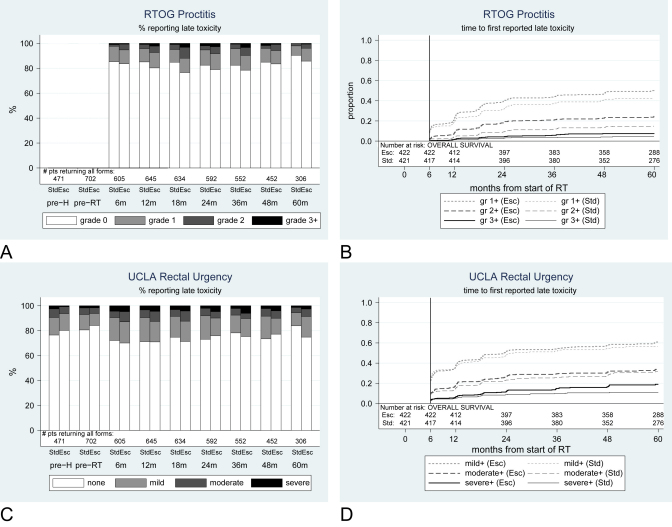
Prevalence and cumulative incidence of proctitis. (A, B) RTOG; (C, D) rectal urgency, UCLA-PCI. Std = standard arm; Esc = escalated arm; m = month; gr = grade; pre-H = prehormonal therapy; pre-RT = preradiation therapy.

Physician-reported proctitis symptoms were rare at baseline, the most common being subjective pain (LENT/SOM); mild symptoms were recorded for 6/702 (1%) patients. Patients reported problems more frequently than doctors, with 76/702 (11%) patients reporting mild rectal urgency at preradiotherapy on UCLA-PCI, 34/702 (5%) patients reporting moderate rectal urgency, and 12/702 (2%) patients reporting severe rectal urgency. On the RTOG scale, mild-to-moderate proctitis was a frequent event during follow-up; significantly more escalated than standard arm patients reported grade ≥2 symptoms during follow-up (HR = 1.64; 95% CI, 1.02-1.52) (Table 3). According to the LENT/SOM subjective scale, cumulative incidence of grade ≥2 tenesmus by 5 years was 13% (51 patients) for the standard arm and 17% (68 patients) for the escalated arm (HR = 1.28; 95% CI, 0.90-1.83). The other LENT/SOM proctitis symptoms were also more frequently reported in the escalated arm at all severities; this difference was statistically significant for grade ≥3 mucosal loss (HR = 2.81; 95% CI, 1.18-6.68) and grade ≥2 sphincter control (HR = 2.42; 95% CI, 1.19-4.89). For pelvic pain (LENT/SOM management of pain), by 5 years, 3 standard and 11 escalated patients required at least regular administration of nonnarcotic analgesics; and 1 standard and 3 escalated patients required regular administration of narcotic analgesics. Medical intervention for mucous discharge and fecal incontinence was rare (LENT/SOM management of sphincter control); in total, by 5 years, 3 standard and 9 escalated patients used pads at least intermittently; 2 and 5 patients, respectively, needed regular pads. There was no significant difference between the treatment arms in terms of mild or moderate rectal urgency (UCLA-PCI) (Fig. 3); however, escalated patients were significantly more likely to report severe symptoms (HR = 1.64; 95% CI, 1.11-2.42).

### Serious bowel injury

Severe side effects associated with older radiotherapy protocols (bowel obstruction, stricture, ulceration) were rarely reported in MRC RT01. Cumulative incidence of rectal ulcers by 5 years was 1% (*n* = 5 patients) in the standard arm and 4% (*n* = 14 patients) in the escalated arm; for rectal stricture, the corresponding proportions were 3% (*n* = 11 patients) and 2% (*n* = 8 patients), respectively. Just 7 standard and 3 escalated patients had reported bowel obstruction by 5 years.

### Patient-reported bowel distress

**Fig. 4:**
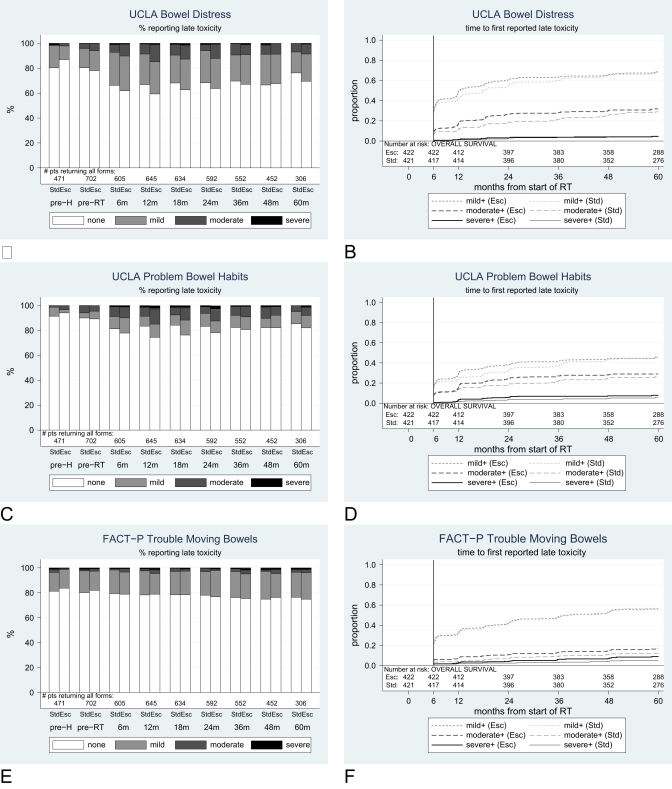
Prevalence and cumulative incidence of patient distress and difficulties. (A, B) Bowel distress, UCLA-PCI; (C, D) problems with bowel habits, UCLA-PCI; (E, F) trouble moving bowels, FACT-P. Std = standard arm; Esc = escalated arm; m = month; gr = grade; pre-H = prehormonal therapy; pre-RT = preradiation therapy.

Preradiotherapy, 109/702 (16%) patients reported mild, 33 (5%) patients reported moderate, and 2 (<1%) patients reported severe distress associated with bowel movements, on the UCLA-PCI questionnaire. Generally, the prevalence of distressing bowel movements and problem bowel habits (UCLA-PCI) at any level increased until 2 or 3 years from starting radiotherapy and then lessened; however, levels were generally still higher than baseline at 5 years in both arms (Fig. 4). By 5 years, the cumulative incidence was 4% (15 patients) in the standard and 5% (16) in the escalated arm for severe distressing bowel movements and 6% (19) and 8% (28) for severe problems with bowel habits (Table 4). There were no statistically significant differences between the arms for these variables, but for all grades, the HR favored the standard arm.

## Discussion

After patients received dose-escalated CFRT for prostate cancer, we found a higher incidence and prevalence of late bowel side effects in the dose-escalated arm after 5 years' follow-up. Rectal bleeding, as measured using the RMH and LENT/SOM objective bleeding scales, peaked at 24 to 36 months after radiotherapy; it then decreased by 5 years on both scales but remained higher than at pretreatment. Regarding the assessment of loose stools and bowel frequency, the RTOG, RMH, and LENT/SOM physician-based scales showed peak late reactions occurring at 12 to 18 months, after which levels returned to near pretreatment levels for the standard arm, with a slight excess for the escalated arm. It is particularly noteworthy that the UCLA-PCI loose stool scale, showing the same general pattern, demonstrates the prevalence and importance of recording baseline bowel disturbance. RTOG proctitis showed a peak at 18 to 36 months and then fell back to pretreatment levels; a similar pattern is seen with UCLA-PCI rectal urgency, with 5-year scores being similar to those at pretreatment. Although many of the scales showed a return to near baseline levels by 5 years, the UCLA bowel distress and problem bowel habit items were still in excess of pretreatment values by 5 years, after peaking at 12 months. The FACT-P “trouble with moving bowels” scale seemed insensitive to treatment effects. More detailed analysis of the interrelationship and correlations between the different scoring schemes will be presented in a future publication.

Like other trials, severe late side effects were very rare (<1%) overall but did occur. Severe rectal bleeding, tenesmus, and rectal ulceration were more common in the dose-escalated group. By 5 years, 4% of standard and 5% of escalated arm patients had reported severe distress at some time because of altered bowel habits.

In this trial, we trained clinicians at different centers to use the same outlining techniques, and we used standardized planning techniques and employed a quality assurance program to minimize differences between treatment centers (6, 7). However, at planning, we did not limit the dose to the rectum, anal canal, or small bowel in either treatment arm; anatomical differences might therefore also contribute to normal tissue toxicity (14–18). We used portal imaging of pelvic bones but not other image-guided techniques; changes in prostate position and rectal volume during treatment were also likely to influence dose to normal tissues and toxicity (19–21). We used three clinician scoring systems (5, 10, 11) and two patient quality of life questionnaires (12, 13). Compliance from clinicians and patients was generally good. Here, we used data only where all assessments were completed for a certain time point, so the data quality is high. As demonstrated, the scoring systems give different details and slightly different toxicity levels. We had to adjust the LENT/SOM scale downward for most bowel questions (but not diarrhea) to allow comparison with original RTOG and RMH scales. For future trials, further efforts to standardize reporting of late side effects are required (22–26).

Allowing for the differences in radiation dose, treatment techniques, toxicity scales, and follow-up time, our results are comparable with results of four published, randomized dose escalation trials (Table 5) (1, 2, 4, 5). Across these trials, the increase in RTOG grade ≥2 toxicity varied, perhaps reflecting differences in dose increments between the standard and escalated schedules (8 Gy and 10 Gy), planning techniques which included initial conventional planning (1), full conformal therapy throughout (2, 5) or proton beam boost (4), toxicity scales, and reporting time points. Although each trial demonstrated improvements in biochemical control, patients with prostate cancer may rate quality of life and freedom from troublesome side effects as important as cancer control, and a significant proportion might choose lower radiation doses because of the worries of late side effects (27, 28). These results should encourage clinicians to discuss with patients the balance of expected benefits and risks of treatment. Future research might usefully assess the contribution of improved dose distributions and normal tissue sparing using intensity-modulated radiation therapy and image guidance to reduce treatment margins (29), the application of appropriate dose constraints (30), and perhaps focal radiation boosts (31) with the aim of maintaining the benefits of disease control with dose escalation while reducing late side effects of treatment.

## Conclusions

Dose-escalated radiotherapy is associated with a statistically significant increased risk of rectal bleeding, which decreases over time. The cumulative incidence by 5 years for moderate-or-worse bleeding was higher in the escalated-dose arm. Diarrhea and proctitis were less common than bleeding. Worrying historical side effects (obstruction, ulceration, and fistulas) were very rare. Patient-reported bowel distress occurred uncommonly, and many toxicities reported seemed not to greatly trouble patients. Additional improvements in radiotherapy technique might maintain the benefits of dose escalation yet further improve patient acceptability.

## Figures and Tables

**Table 1:** Cumulative incidence and risk of rectal bleeding

			Cumulative incidence at 5 years			
			64 Gy		74 Gy	
Symptom: scale	HR	95% CI	*n* patients	% of patients	*n* patients	% of patients
Rectal bleeding: RMH						
Mild	1.32	1.08–1.61	170	43	111	53
Moderate	1.97	1.37–2.84	43	11	81	21
Severe	1.65	0.39–6.92	3	1	5	1
Objective bleeding: LENT/SOM						
Grade ≥2	1.55	1.17–2.04	83	22	121	32
Grade ≥3	3.12	1.63–6.01	12	3	36	10
Grade ≥4	0.68	0.11–4.04	3	1	1	1
Management bleeding: LENT/SOM						
Grade ≥2	1.54	0.74–3.20	9	3	17	4
Grade ≥3	3.07	0.83–11.33	3	1	8	2
Grade ≥4	5.12	0.60–43.8	1	0	4	1

**Table 2:** Cumulative incidence and risk of diarrhea and frequency

			Cumulative incidence at 5 years			
			64 Gy		74 Gy	
Symptom: scale	HR	95% CI	n patients	% of patients	n patients	% of patients
Diarrhea: RTOG						
Grade ≥1	1.06	0.84-1.34	130	33	137	34
Grade ≥2	1.59	1.07-2.35	38	10	60	15
Grade ≥3	3.04	1.21-7.67	5	1	16	4
Bowel frequency: RMH						
Mild	1.12	0.89-1.41	139	32	144	36
Moderate	1.38	0.92-2.07	35	9	54	14
Severe	2.23	1.09-4.55	9	2	24	6
Subjective stool frequency: LENT/SOM						
Grade ≥1	1.07	0.87-1.32	172	44	181	46
Grade ≥2	1.79	1.10-2.94	23	6	43	12
Grade ≥3	NA	NA	0	0	9	2
Management tenesmus: LENT/SOM						
Grade ≥2	1.42	0.83-2.42	6	23	8	32
Grade ≥3	2.28	0.70-7.41	1	4	2	9
Grade ≥4	NA	NA	0	0	0	0
Loose stools: UCLA-PCI						
Mild	1.14	0.98-1.31	360	91	378	94
Moderate	1.25	0.90-1.73	66	17	77	21
Severe	0.95	0.51-1.75	21	6	20	6
Pain abdomen: UCLA-PCI						
Mild	1.19	0.98-1.44	193	51	223	58
Moderate	1.53	1.13-2.06	71	21	103	28
Severe	1.55	0.84-2.86	17	5	26	7

*Abbreviation:* NA = not applicable.

**Table 3:** Cumulative incidence and risk of proctitis

			Cumulative incidence at 5 years			
			64 Gy		74 Gy	
Symptom: scale	HR	95% CI	*n* patients	% of patients	*n* patients	% of patients
Proctitis: RTOG						
Grade ≥1	1.24	1.02–1.52	167	42	199	50
Grade ≥2	1.64	1.20–2.25	58	15	96	25
Grade ≥3	1.74	0.97–3.10	17	4	29	7
Subjective tenesmus: LENT/SOM						
Grade ≥2	1.28	0.90–1.83	51	13	68	17
Grade ≥3	1.75	0.90–3.38	14	4	23	6
Grade ≥4	NA	NA	0	0	1	0
Subjective mucosal loss: LENT/SOM						
Grade ≥2	1.31	0.78–2.21	24	6	31	8
Grade ≥3	2.81	1.18–6.68	6	2	18	5
Grade ≥4	NA	NA	0	0	1	0
Subjective sphincter control: LENT/SOM						
Grade ≥2	2.42	1.19–4.89	10	5	25	7
Grade ≥3	9.25	1.17–73.02	1	0	9	2
Grade ≥4	NA	NA	0	0	1	0
Subjective rectal pain: LENT/SOM						
Grade ≥2	1.46	0.84–2.52	21	5	31	8
Grade ≥3	7.14	0.88–58.01	1	0	7	2
Grade ≥4	NA	NA	0	0	0	0
Management sphincter control: LENT/SOM						
Grade ≥2	3.04	0.82–11.22	3	1	9	2
Grade ≥3	2.54	0.49–13.07	2	0	5	1
Grade ≥4	NA	NA	0	0	0	0
Management pelvic pain: LENT/SOM						
Grade ≥2	3.76	1.05–13.47	3	1	11	3
Grade ≥3	3.07	0.32–29.53	1	0	3	1
Grade ≥4	NA	NA	0	0	1	0
Rectal urgency: UCLA-PCI						
Mild	1.06	0.88–1.27	119	57	231	61
Moderate	1.11	0.86–1.42	114	32	125	34
Severe	1.64	1.11–2.42	41	11	66	19

**Table 4:** Cumulative incidence of difficulties with bowel habits from patient questionnaires

			Cumulative incidence at 5 years			
			64 Gy		74 Gy	
Symptom: scale	HR	95% CI	*n* patients	% of patients	*n* patients	% of patients
Bowel distress: UCLA-PCI						
Mild	1.08	0.91–1.29	258	68	268	61
Moderate	1.26	0.96–1.64	101	29	119	32
Severe	1.07	0.53–2.17	15	4	16	5
Bowel problems: UCLA-PCI						
Mild	1.09	0.89–1.35	167	46	175	46
Moderate	1.21	0.92–1.59	96	26	110	29
Severe	1.49	0.83–2.67	19	6	28	8
Trouble moving bowel: FACT-P						
Mild	1.01	0.83–1.22	211	57	210	56
Moderate	1.39	0.94–2.05	42	12	59	17
Severe	1.63	0.91–2.94	17	5	29	9

**Table 5:** Cumulative incidence of late RTOG grade ≥2 toxicity in five randomized trials

	Trial (ref.[s])				
Toxicity	M. D. Anderson (1)	NKI (2, 3)	PROG 9509 (4)	RMH pilot (5)	MRC RT01 (6, 7)
RT dose Gy	70 vs. 78	68 vs. 78	70.2 vs 79.2	64 vs. 74	64 vs. 74
Setting	US	The Netherlands	US	UK	UK, Australia, New Zealand
Sites	Single site	Multisite	Single site	Single site	Multisite
RT technique	CFRT photon	CFRT photon	CFRT photon	CFRT photon	CFRT photon
	CFRT boost	CFRT boost	Proton boost	CFRT boost	CFRT boost
No. of patients randomized	301	669	393	126	843
Toxicity scale	RTOG-LENT modified∗	RTOG/EORTC	RTOG	RTOG original	RTOG original
Median follow-up (years)	8.7	5.8	5.5	6.2	5.3
Grade ≥2 64 Gy vs. 74 Gy	13% vs. 26% (*p* = 0.013)	25% vs. 35% (*p* = 0.04)	9% vs. 18% (*p* = 0.005)	11% vs. 23% (*p* = 0.02)	24% vs. 33% (*p* = 0.005)
Analysis time point and type	By 10 years cumulative	By 7 years cumulative	“Late” snapshot	By 2 years cumulative	By 5 years cumulative

∗ Composite score including rectal bleeding.

## Supplementary Data

Table E1
