# Supplementary material for: Late Gastrointestinal Toxicity After Dose-Escalated Conformal Radiotherapy for Early Prostate Cancer: Results From the UK Medical Research Council RT01 Trial (ISRCTN47772397)
Source: Int J Radiat Oncol Biol Phys. 2010 Jul 1;77(3-2):773–83. doi: 10.1016/j.ijrobp.2009.05.052 (PMC2937212; doi:10.1016/j.ijrobp.2009.05.052)
Supplement: Table E1 [file mmc1.doc]

Table e1: Categorisation of toxicity assessments

|  | | **Original category** | **Category in analyses** |
| --- | --- | --- | --- |
| **RTOG*** | |  |  |
| Diarrhoea, Proctitis, Bowel obstruction, Rectal ulcer, Rectal stricture | | Grade 0 | Grade 0 |
|  |  | Grade 1 | Grade 1 |
|  |  | Grade 2 | Grade 2 |
|  |  | Grade 3 | Grade 3+ |
|  |  | Grade 4 | Grade 3+ |
|  |  | Grade 5 | Grade 3+ |
| **RMH†** | |  |  |
| Bowel frequency | | 1-2 times | None |
|  |  | 3-4 times (no medical treatment) | Mild |
|  |  | 3-4 times (simple OPM‡) | Moderate |
|  |  | ≥ 5 times with or without treatment | Severe |
| Rectal bleeding | | None | None |
|  |  | Occasional no treatment | Mild |
|  |  | Moderate (simple OPM) | Moderate |
|  |  | Severe (blood transfusion, surgery) | Severe |
| **LENT/SOM§** | |  |  |
| Subjective | Tenesmus, Mucosal loss, Sphincter control, Pain | Grade 0 | Grade 0-1 |
|  |  | Grade 1 | Grade 0-1 |
| Objective | Bleeding | Grade 2 | Grade 2 |
| Management | Tenesmus & stool frequency, Bleeding, Sphincter control, Pain | Grade 3 | Grade 3 |
|  |  | Grade 4 | Grade 4 |
|  |  |  |  |
| Subjective stool frequency | | Grade 0 | Grade 0 |
|  |  | Grade 1 | Grade 1 |
|  |  | Grade 2 | Grade 2 |
|  |  | Grade 3 | Grade 3-4 |
|  |  | Grade 4 | Grade 3-4 |
| **FACT-P||** | |  |  |
| I have trouble moving my bowels | | Not at all | None |
|  |  | A little bit | Mild |
|  |  | Somewhat | Mild |
|  |  | Quite a bit | Moderate |
|  |  | Very much | Severe |
| **UCLA PCI¶** | |  |  |
| How often have you had rectal urgency? | | Rarely or never | None |
|  |  | About once a week | Mild |
|  |  | More than once a week | Mild |
|  |  | About once a day | Moderate |
|  |  | More than once a day | Severe |
| How often have you had stools that were loose or liquid during the last 3 weeks? | | Never | None |
|  |  | Rarely | Mild |
|  |  | About half the time | Mild |
|  |  | Usually | Moderate |
|  |  | Always | Severe |
| How much distress have your bowel movements caused you during the last 4 weeks? | | No distress | None |
|  |  | Little distress | Mild |
|  |  | Moderate distress | Moderate |
|  |  | Severe distress | Severe |
| How often have you had crampy pain in your abdomen or pelvis during the last 4 weeks? | | Rarely or never | None |
|  |  | About once this month | Mild |
|  |  | About once a week | Mild |
|  |  | Several times a week | Moderate |
|  |  | About once a day | Moderate |
|  |  | Several times a day | Severe |
| Overall, how big a problem have your bowel habits been for you during the last 4 weeks? | | No problem | None |
|  |  | Very small problem | None |
|  |  | Small problem | Mild |
|  |  | Moderate problem | Moderate |
|  |  | Big problem | Severe |

*Abbreviations:* *RTOG = Radiation Therapy Oncology Group Scale; †RMH = Royal Marsden score; ‡OPM = outpatient management; §LENT/SOM = Late Effects on Normal Tissue scale; **||**FACT-P = Functional Assessment of Cancer Therapy-Prostate; ¶UCLA PCI = University of California, Los Angeles Prostate Cancer Index.
